# Supplementary material for: Where Are We Now? Feeds, Feeding Systems and Current Knowledge of UK Horse Owners When Feeding Haylage to Their Horses
Source: Animals (Basel). 2023 Apr 7;13(8):1280. doi: 10.3390/ani13081280 (PMC10135377; doi:10.3390/ani13081280)
Supplement: Supplementary file 1 [file animals-13-01280-s001.zip › Survey 1.pdf]

Question 1: What age are you?

15-25
26-30
31-40
41-50
51+

Question 2: What equine related education have you had? (tick as many as apply)

None
Industry experience
Pony club/Riding club
BHS Qualifications
Diploma/Degree
Masters/PHD

Question 3: How many horses do you have in each age bracket? (tick as many as apply) For the following questions, if you have more than one horse and treat them differently please tick the number of horses that each answer relates to, if you only have one then just tick 1 to which ever answer you chose.

Age	Number of horses				
	1	2	3	4	5+
0-5 years					
6-14 years					
15-20 years					
21+ years					

Question 4: How big is/are your horse/s?

Height (hh)	Number of horses				
	1	2	3	4	5+
Small <14.2					
Medium 14.2-16.2					
Large >16.2					

Question 5:
Is/are your horse/s currently stabled?

Amount of time	Number of horses				
	1	2	3	4	5+
During/part of the day					
At night					
All the time					
Never					

Question 6: How many times a day do you feed meals to your horse/s?

Number of meals a day	Number of horses				
	1	2	3	4	5+
Never					
Once					
Twice					
3 times					
4+ times					

Question 7: How often is/are you horse/s ridden, lunged, worked or exercised a week?

Number of times a week	Number of horses				
	1	2	3	4	5+
Retired/pet					
1-2 times					
3-5 times					
6-7 times					

Question 8: What is your main discipline?

Hacking and pleasure riding
Showjumping
Dressage
Eventing
Polo
Racing
Hunting
Western
Driving
Showing
Other (please specify)

Question 9: Which forage do you feed your horse/s?

Hay
Haylage
Silage
Other (please specify)

Question 10: Do you weigh out your forage?

Yes
No

10 a. If yes, how much?

0-5kg
5-10kg
10-15kg+

Question 11: How do you feed your forage?

- Net
- Floor
- Hay bar/rack
- Other (please specify)

Question 12: Have you ever had your forage analysed?

- Yes
- No

12 a. If no, why not?

- Have not heard about forage analysis
- Don't see a need for forage analysis
- Haven't got around to it/time limitations
- Cost
- Yard provides forage
- Other (please specify)

Question 13: What fibre feed do you feed your horse/s?

- Chaff
- Alfalfa
- Sugar beet
- Complete chaff feed
- Fast fibre
- None
- Other (please specify)

Question 14: Do you measure out your fibre feed?

- Yes
- No
- Don’t feed one

14 a. If yes, how?

- Round scoop
- Square scoop
- Accurate scales measurement
- By eye
- Handfuls
- Other (please specify)

Question 15: What concentrate do you feed your horse/s?

- Cereals (oats/barley/maize, whole or rolled)
- High fibre nuts
- Cereal based course mix
- Compound nuts
- Grass nut
- Rice bran

Balancer
None
Other (please specify)

Question 16: Do you measure you concentrate?
Yes
No
Don't feed one

16 a. If yes, how?
Round scoop
Square scoop
Accurate scales measurement
By eye
Handfuls
Other (please specify)

Question 17: Do you stick to a particular brand of fibre feed?
Yes
No

17a. If yes, please rank the following reasons as to why, with 1 being the most important and 7 being the least.

Reason	Ranking						
	1	2	3	4	5	6	7
Loyalty							
Sponsorship							
Price							
Feed composition							
Nutritional advice							
Keep the feed regular for your horse							
Feed works well for your horse							

Question 18: Do you stick to a particular brand of concentrate feed?
Yes
No

18 a. If yes, please rank the following reasons as to why, with 1 being the most important and 7 being the least.

Reason	Ranking						
	1	2	3	4	5	6	7
Loyalty							
Sponsorship							

Price							
Feed composition							
Nutritional advice							
Keep the feed regular for your horse							
Feed works well for your horse							

Question 19: Do you seek nutritional advice prior to purchasing any form of horse feed? (tick as many as apply)

Family
Friends
Nutritionist
Instructor
Yard manager
Vet
Internet forms
None

Question 20: Do you feed supplements to you horse/s?

Yes
No

20 a. If yes, why?

Prevention
Health based condition
Advertisements make products look attractive
Balance the diet
Other (please specify)

20 b. Do you seek advice on what supplements to feed?

Family
Friends
Nutritionist
Instructor
Yard manager
Vet
Internet forms
None

Question 21: Do you feel your feeding practices have changed?

Yes, over the last 5 years
Yes, over the last 5-10 years
Yes, over the last 10+ years
Not changed

21 a. Please rank why have your feeding practices changed from most important (1) to least important (7)

Reason	Ranking						
	1	2	3	4	5	6	7
More education/research available							
Wider range of feed							
Less time available to spend with horse							
Cost							
Different horses' needs							
Higher competition level/more energy needs of the horse							
Convenience of getting the feed							

21 b. How have your feeding practices changed?

More forage
Less forage
More concentrate
Less concentrate
Different feed types
Other (please specify)

Please add any additional comments you may have

Thank you for taking part in my survey
