# Supplementary material for: Where Are We Now? Feeds, Feeding Systems and Current Knowledge of UK Horse Owners When Feeding Haylage to Their Horses
Source: Animals (Basel). 2023 Apr 7;13(8):1280. doi: 10.3390/ani13081280 (PMC10135377; doi:10.3390/ani13081280)
Supplement: Supplementary file 1 [file animals-13-01280-s001.zip › Survey 2.pdf]

Haylage survey

Introduction

You are being invited to participate in a research study into the use of haylage as a forage source for horses in the UK. This study is being conducted by Lucile Rousson, Sue Martin, Simon Daniels and Meriel Moore-Colyer from the Royal Agricultural University and Pat Harris from the WALTHAM Equine Studies Group.

The purpose of this research study is to gain insight into the use of haylage, buying patterns and perceptions of this forage source. We are interest in your opinions and current knowledge, so please answer using the most appropriate option(s) to your beliefs or normal practices when feeding horses. We appreciate that many of you may have multiple horses that are managed in different ways, when answering the questions please answer with the most typical or most common ways that you feed your horses. We are interested in your response therefore please do not be tempted to look up answers online, please answer to the best of your knowledge or reflecting on what you do rather than what you think you should do.

The survey will take you approximately 10 minutes to complete. Your participation in this study is entirely voluntary, all data are totally anonymous therefore once the questionnaire has been sent data cannot be identified to be removed. By submitting your results you are therefore agreeing to the use of your data in this study.

This survey has been approved by the Royal Agricultural University Research Ethics committee (project approval number: 2020.0040). If you have any questions, please do not hesitate to contact Dr Simon Daniels via e-mail simon.daniels@rau.ac.uk

General knowledge about haylage

In this first part, we really want to know your genuine opinions and knowledge of haylage. Please do not search the internet to answer questions.

1. Which of these best describes haylage? Please choose up to 2 answers ✱ *Required*

Please select between 1 and 2 answers.

- ☐ Wrapped fermented grass
- ☐ Wrapped partially fermented grass
- ☐ Wrapped dried grass
- ☐ Wrapped partially dried grass
- ☐ Wrapped wet grass
- ☐ Late cut wrapped grass
- ☐ Early cut wrapped grass

2. How does haylage typically differ from hay? Please choose up to 3 answers ✱ *Required*

Please select between 1 and 3 answers.

- ☐ Higher dust than hay
- ☐ Lower dust than hay
- ☐ Higher nutritional content than hay
- ☐ Lower nutritional content than hay
- ☐ Softer than hay
- ☐ Sweeter smelling than hay
- ☐ Higher sugar content (Water Soluble Carbohydrates(WSC)) than hay
- ☐ Lower sugar content than hay (WSC)
- ☐ More acidic than hay
- ☐ Less acidic than hay
- ☐ I don't know the difference

3. Do you feed your horse(s) haylage? ✱ *Required*

- ○ Yes
- ○ No

3.a. If you do not feed haylage, why? Please choose up to 3 answers

Please select between 1 and 3 answers.

- ☐ Energy content too high
- ☐ Problems with bale size/storage
- ☐ I prefer hay

- [ ] Horse(s) gain too much weight
- [ ] Too high in protein
- [ ] Horse(s) display excitable behaviour
- [ ] More expensive than hay
- [ ] It is not available where I keep my horse(s)
- [ ] Concerns about dental problems
- [ ] Concerns about gastric ulcers
- [ ] Other

3.a.i. If you selected Other, please specify:

3.b. Do you feed both hay and haylage to at least one horse?

- ○ Yes
- ○ No

3.b.i. How many horses do you feed both with hay and haylage?

- ○ All horses (more than one)
- ○ Majority of horses (more than one)
- ○ Individual horse (where you have more than one)
- ○ One horse (you only have one)

3.b.i.a. Why do you not feed all of your horses hay and haylage?

3.b.i.b. Why do you feed an individual horse both hay and haylage?

3.b.ii. Which is the closest ***most common*** approximate ratio of hay to haylage you ***typically*** feed? e.g : 30:70 means 30% of haylage and 70% of hay

○ 10:90	○ 20:80	○ 30:70
○ 40:60	○ 50:50	○ 60:40
○ 70:30	○ 80:20	○ 90:10

3.b.iii. If you feed both hay and haylage can you give up to 2 reasons why?

Please select between 1 and 2 answers.

- [ ] More cost effective to feed both
- [ ] To provide enough fibre by combining the two
- [ ] To manage weight loss/gain
- [ ] To balance the diet
- [ ] To provide variety in the diet
- [ ] Dental recommendation
- [ ] Other

3.b.iii.a. If you selected Other, please specify:

3.b.iv. When feeding haylage alone would you feed :

- ( ) The same weight as you would for hay
- ( ) A greater weight than if feeding hay
- ( ) Less than if feeding hay

3.b.iv.a. Why do you feed the same weight of haylage as you would for hay? Please choose your top 2 main reasons.

Please select between 1 and 2 answers.

- [ ] They are interchangeable forage sources
- [ ] Haylage is likely to have a similar nutritional content to hay
- [ ] I don't want my horse to gain weight
- [ ] I want my horse to lose weight
- [ ] I like variety in the diet
- [ ] I want to avoid excitable behaviour
- [ ] No specific reason
- [ ] Other

3.b.iv.a.i. If you selected Other, please specify:

3.b.iv.b. Why do you feed less haylage than hay? Please choose your top 2 main reasons.

Please select between 1 and 2 answers.

- [ ] Haylage has a higher energy content than hay
- [ ] Higher in sugar (WSC) than hay
- [ ] To reduce the risk of gastric ulcers
- [ ] To reduce the risk of laminitis
- [ ] To prevent weight gain
- [ ] Haylage provides more fibre than hay
- [ ] Haylage provide more nutrient but less bulk than hay

- [ ] No specific reason
- [ ] Other

3.b.iv.b.i. If you selected Other, please specify:

3.b.iv.c. Why do you feed more haylage than hay? Please choose your top 2 main reasons.

Please select between 1 and 2 answers.

- [ ] Haylage has a greater moisture content
- [ ] To reduce the risk of gastric ulcers
- [ ] To reduce the risk of laminitis
- [ ] To promote weight gain
- [ ] Haylage provides more fibre than hay
- [ ] Haylage provide more nutrient but less bulk than hay
- [ ] No specific reasons
- [ ] Other

3.b.iv.c.i. If you selected Other, please specify:

Buying and storage pattern

4. Where do you get most of your haylage from? ✱ *Required*

- Make my own
- Make my own and buy in additional from a local farmer
- Make my own and buy in additional from a merchant (hay/haylage supplier)
- Make my own and buy in additional from a commercial haylage producer
- Buy from a local farm
- Buy from a haylage merchant (hay/haylage supplier)
- Buy from a commercial haylage producer

4.a. What percentage of the haylage you use per year do you buy in?

- 10%
- 20%
- 30%
- 40%
- 50%
- 60%
- 70%
- 80%
- 90%

4.b. When do you buy haylage?

- Throughout the year
- Before making my own
- When I run out
- In winter
- Other

4.b.i. If you selected Other, please specify:

4.c. When sourcing haylage for the year do you buy :

- Enough for the year at once
- Every six months
- Every three months
- Monthly
- Weekly
- When I run out
- Other

4.c.i. If you selected Other, please specify:

4.d. When sourcing haylage for the year do you buy :

- Enough for the year at once
- Every six months
- Every three months
- Monthly
- Weekly
- When I run out
- Other

4.d.i. If you selected Other, please specify:

____

4.d.ii. If you buy more than once per year, does all your haylage come from the same field cut?

- Yes
- No
- Don't know

4.e. When sourcing haylage for the year do you buy :

- Enough for the year at once
- Every six months
- Every three months
- Monthly
- Weekly
- When I run out

4.e.i. If you buy more than once per year, does all your haylage come from the same farm?

- Yes
- No
- Don't know

4.f. What sized bales do you **mainly** make?

- Big (square or round, approx. 200-300kg)
- Medium size (approx 125kg)
- Small (approx. 20kg)

4.f.i. Why do you choose this size? Please, give up to 2 reasons

Please select between 1 and 2 answers.

- [ ] I have several horses
- [ ] I have one horse
- [ ] It is easier to store

- [ ] I don't have a choice on bale size
- [ ] Most economic option
- [ ] Other

4.f.i.a. If you selected Other, please specify:

4.g. What sized bales do you mainly buy?

- ( ) Big (square or round, approx. 200-300kg)
- ( ) Medium size (approx 125kg)
- ( ) Small (approx. 20kg)

4.g.i. Why do you choose this size? *Optional*

- ( ) I have several horses
- ( ) I have one horse
- ( ) It is easier to store
- ( ) This is the bale size provided where I keep my horse
- ( ) Most economic
- ( ) Other

4.g.i.a. If you selected Other, please specify:

4.h. Why do you buy from a commercial haylage producer? Please choose your top 3 answers

Please select exactly 3 answer(s).

- [ ] Quality
- [ ] More convenient
- [ ] Easier to store small bales
- [ ] Easier to transport small bales
- [ ] Comes with a feed analysis
- [ ] Consistency
- [ ] Other

4.h.i. If you selected Other, please specify:

5. How many horses do you own/keep, and how many are kept at your premises?

	1	2	3	4	5	6	7	8	9	10+
Number of horses I own/manage										
Number of horses on the premises										

6. Typically, how are your horses kept throughout the year?
By "stabled", we mean any indoor system
By "field", we mean any outdoor system

Please don't select more than 4 answer(s) per row.

	Winter	Spring	Summer	Autumn
Mainly stabled most of the time				
Mainly field kept most of the time				
A combination of stabling and field turnout				

7. When starting a new bale from the same cut, do you : ✱ *Required*

- Gradually introduce the new bale
- Move directly from one to another within a day
- Other

7.a. If you selected Other, please specify:

7.b. Over how many days would you make the change?

- 2-3 days
- 4-5 days
- 6+ days

7.c. Approximately for a typical horse, how frequently do you open a new haylage bale? ✱ *Required*

- 2-3 days
- 4-5 days
- 6+ days
- Other

7.c.i. If you selected Other, please specify:

8. When changing to one bale from another from a **different field cut or supplier**, do you : ✱ *Required*

- Gradually introduce the new bale
- Move directly from one bale to another
- Other

8.a. If you selected Other, please specify:

8.b. Over how many days would you make the change?

- 2-3 days
- 4-5 days
- 6+ days

9. When changing to one bale from another from a **different year's crop**, do you : ✱ *Required*

- Gradually introduce the new bale
- Move directly from one to another within a day
- Other

9.a. If you selected Other, please specify:

9.b. Over how many days would you make the change?

- 2-3 days
- 4-5 days
- 6+ days

What do you view as good quality haylage?

10. What do you look for when opening a bale to determine the quality of the haylage? Please rank at least 4 of the following answers with 1 being your most important sign of good quality. You may choose up to 11 if you wish ✱ *Required*

Please don't select more than 1 answer(s) per row.

Please select between 4 and 11 answers.

Please don't select more than 1 answer(s) in any single column.

	1	2	3	4	5	6	7	8	9	10	11
Soft touch	☐	☐	☐	☐	☐	☐	☐	☐	☐	☐	☐
No dust when I shake	☐	☐	☐	☐	☐	☐	☐	☐	☐	☐	☐
Sweet smell	☐	☐	☐	☐	☐	☐	☐	☐	☐	☐	☐
Quite moist	☐	☐	☐	☐	☐	☐	☐	☐	☐	☐	☐
No visible sign of mould	☐	☐	☐	☐	☐	☐	☐	☐	☐	☐	☐
Strong acidic smell	☐	☐	☐	☐	☐	☐	☐	☐	☐	☐	☐
Lots of leaf	☐	☐	☐	☐	☐	☐	☐	☐	☐	☐	☐
Lots of stem	☐	☐	☐	☐	☐	☐	☐	☐	☐	☐	☐
Strong green colour	☐	☐	☐	☐	☐	☐	☐	☐	☐	☐	☐
Pale green colour	☐	☐	☐	☐	☐	☐	☐	☐	☐	☐	☐
Other	☐	☐	☐	☐	☐	☐	☐	☐	☐	☐	☐

10.a. If you choose other, please tell why :

11. Does your haylage come with an analysis?

- ○ From the farmer/merchant each year
- ○ From the farmer/merchant every time they deliver
- ○ Printed on the packaging of commercial haylage
- ○ No analysis available

11.a. What type of nutritional analysis do they provide?

- ☐ Dry matter
- ☐ Fibre
- ☐ Protein
- ☐ Sugar (WSC)
- ☐ Other

11.a.i. If you selected Other, please specify:

____

12. Do you pay to have your haylage analysed? ✱ *Required*

- ○ Yes
- ○ No

12.a. If yes, how often do you get your haylage analysed?

- ○ For each bale
- ○ For each batch from the same farm
- ○ Every time I buy haylage from a supplier
- ○ Once a year
- ○ When feeding horse(s) with certain clinical problems
- ○ Other

12.a.i. If you selected Other, please specify:

12.a.ii. What type of clinical problem?

12.b. If yes, what do you test for?

- ☐ Hygienic content
- ☐ Nutritional content

12.b.i. What does your hygiene analysis include? Tick all that apply

- ☐ Mould
- ☐ Dust
- ☐ Contaminants (e.g. weeds and soil)
- ☐ Bacteria
- ☐ Other

12.b.i.a. If you selected Other, please specify:

12.b.ii. What does your nutritional analysis include? Tick all that apply

- ☐ Dry matter
- ☐ Energy
- ☐ Protein
- ☐ Sugar (WSC)
- ☐ Minerals
- ☐ Vitamins
- ☐ Fibre
- ☐ Other

12.b.ii.a. If you selected Other, please specify:

12.c. Where do you get your haylage analysed?

- ○ Directly from a laboratory
- ○ Through a feed company

12.c.i. How did you choose the laboratory?

- ○ Advertising
- ○ Recommend by a friend
- ○ Recommend by a feed company
- ○ Recommend by a professional (e.g. a vet, a trainer, a dental technician, a farrier)
- ○ Other

12.c.i.a. If you selected Other, please specify:

What do you think of haylage?

13. What do you feel are **the top three benefits** of feeding haylage? Please rank where 1 is the top benefit, you may rank up to 10 if you wish. ✱ *Required*

Please don't select more than 1 answer(s) per row.

Please select between 3 and 10 answers.

Please don't select more than 1 answer(s) in any single column.

	1	2	3	4	5	6	7	8	9	10
Higher than hay in energy content	☐	☐	☐	☐	☐	☐	☐	☐	☐	☐
Higher than hay for protein content	☐	☐	☐	☐	☐	☐	☐	☐	☐	☐
More palatable	☐	☐	☐	☐	☐	☐	☐	☐	☐	☐
Suitable for horses with gastric ulceration	☐	☐	☐	☐	☐	☐	☐	☐	☐	☐
Suitable for horses with laminitis	☐	☐	☐	☐	☐	☐	☐	☐	☐	☐
Suitable for horses with respiratory conditions	☐	☐	☐	☐	☐	☐	☐	☐	☐	☐
Suitable for horses with dental problems	☐	☐	☐	☐	☐	☐	☐	☐	☐	☐
Good for weight gain	☐	☐	☐	☐	☐	☐	☐	☐	☐	☐
Lower in sugar than hay	☐	☐	☐	☐	☐	☐	☐	☐	☐	☐
Other	☐	☐	☐	☐	☐	☐	☐	☐	☐	☐

13.a. If you selected Other, please specify:

14. What do you feel are the **top three disadvantages** of feeding haylage? Please rank where 1 is the top disadvantage, you may rank up to 11 if you wish.

Please don't select more than 1 answer(s) per row.

Please select between 3 and 11 answers.

	1	2	3	4	5	6	7	8	9	10	11
Horses gain excessive weight	☐	☐	☐	☐	☐	☐	☐	☐	☐	☐	☐
Horse(s) show excitable behaviour	☐	☐	☐	☐	☐	☐	☐	☐	☐	☐	☐
Not suitable for horses with gastric ulcers	☐	☐	☐	☐	☐	☐	☐	☐	☐	☐	☐
Not suitable for horses with laminitis	☐	☐	☐	☐	☐	☐	☐	☐	☐	☐	☐
Not suitable for horses with dental problems	☐	☐	☐	☐	☐	☐	☐	☐	☐	☐	☐
Rapid aerobic spoilage	☐	☐	☐	☐	☐	☐	☐	☐	☐	☐	☐
Not sure how much to feed (more or less than hay)	☐	☐	☐	☐	☐	☐	☐	☐	☐	☐	☐
Available bale sizes are not suitable	☐	☐	☐	☐	☐	☐	☐	☐	☐	☐	☐
Too palatable	☐	☐	☐	☐	☐	☐	☐	☐	☐	☐	☐
Cost	☐	☐	☐	☐	☐	☐	☐	☐	☐	☐	☐
Other	☐	☐	☐	☐	☐	☐	☐	☐	☐	☐	☐

14.a. If you selected Other, please specify:

____

15. Which country are you from? ✱ *Required*

- ○ Belgium
- ○ Denmark
- ○ Ireland
- ○ Netherlands
- ○ United Kingdom

- Other

15.a. If you selected Other, please specify:

15.b. Where do you live?

- North of England
- South of England
- East of England
- West of England
- Wales
- Scotland
- Northern Ireland
- Midlands

Thank you

Thank you for your time and your answers

End
